# Efficacy and evaluation of dose-response relationship of selective internal radiation therapy for the management of liver metastases in neuroendocrine neoplasia

**DOI:** 10.1007/s00259-026-07762-0

**Published:** 2026-02-17

**Authors:** Qasim Ahmed, Nikolaos Doumanoglou, Joanne Evans, Maria Martinez, Caroline Ward, Hooshang Izadi, Florian Wernig, Mark Bray-Parry, Kuldip Nijran, Laura Perry, Neva Patel, Chloe Bowen, Ali Alsafi, Rob Thomas, Tahir Shah, Priten Khagram, Paul Tait, Rohini Sharma

**Affiliations:** 1 Division of Surgery and Cancer, Imperial College London, Hammersmith Hospital, Du Cane Road, London , W12 0NN UK; 2 School of Engineering, Computing and Mathematics, Oxford Brookes University, Oxford, UK; 3 Department of Endocrinology, Imperial College NHS Healthcare Trust, Hammersmith Hospital, Du Cane Road, London, W12 0HS UK; 4 The London Clinic, 20 Devonshire Place, London, W1G 6JA UK; 5 Nuclear Medicine Department, Imperial College NHS Healthcare Trust, Hammersmith Hospital, Du Cane Road, London, W12 0HS UK; 6 Department of Interventional Radiology, Imperial College NHS Healthcare Trust, Hammersmith Hospital, Du Cane Road, London, W12 0HS UK; 7 National Institute for Health Research Birmingham Liver Biomedical Research Unit and Centre for Liver and Gastrointestinal Research, Institute of Immunology and Immunotherapy, University of Birmingham, Birmingham, UK

**Keywords:** Radioembolisation, Neuroendocrine neoplasia, Liver metastases, Survival, Yttrium

## Abstract

**Purpose:**

The liver is the commonest site of metastases in neuroendocrine neoplasias (NENs) and is an independent predictor of poor outcome. Selective internal radiation therapy (SIRT) allows selective delivery of high dose radiation to liver tumours and has shown promise in the management of NENs. We determined the safety and efficacy of SIRT for inoperable liver metastases secondary to NENs. Dose-response and dose-toxicity relationship were assessed.

**Methods:**

A prospective, multicentre, phase 2 study was conducted. Primary outcomes were objective response rate (ORR) in the treated liver volume according to RECIST 1.1 and mRECIST criteria, incidence and severity of adverse events (AEs) at 6 months. Secondary outcomes were hepatic specific progression free survival, PFS, overall survival (OS), change in quality of life (QoL). Tumour dose-response relationship was derived retrospectively from post therapy yttrium-90 bremsstrahlung single photon emission computed tomography/CT for both the tumour and perfused normal liver.

**Results:**

21 patients were analysed; majority had grade 2 NEN (67%) and were heavily pretreated, 76% having received prior systemic therapy including peptide receptor radiotherapy (33%). ORR by RECIST 1.1 and mRECIST were 14% and 45%, respectively. Median hepatic specific PFS, PFS and OS were 48.1, 13.3 and 49.9 months respectively. Only 3 patients experienced grade 3 AEs; 2 radiation-induced liver disease that resolved without sequelae and one arterial plug migration. No significant deterioration in QoL was observed following SIRT. Dosimetry analysis found a clear tumour dose/response relationship for 3-month ORR. Mean tumour dose in responders was 372 Gy versus 173 Gy in non-responders (*p* < 0.001). We identified a threshold tumour absorbed dose of 164 Gy for PFS 48.1months compared to 8.8months (HR 0.2, 95%CI 0.05–0.96. *p* = 0.03).

**Conclusion:**

In a prospective study of heavily pretreated patients, we have demonstrated clinical efficacy of SIRT for the management of liver metastases secondary to NENs. A tumour dose-response relationship was demonstrated and a threshold tumour dose for survival outcome. This work lends prospective evidence for personalised dosimetry in NENs.

**Supplementary information:**

The online version contains supplementary material available at 10.1007/s00259-026-07762-0.

## Introduction

Neuroendocrine neoplasia (NENs) are a heterogenous cancer type that arises from the cells of the neuroendocrine system. Most NENs present with vague symptomology and as a result, up to 75% of patients will present with metastatic disease, the commonest site being the liver [1, 2]. The presence of metastatic disease per se confers a 10-fold increase in mortality risk [3], with hepatic metastases being a powerful independent prognostic factor. There is therefore considerable interest in investigating liver directed therapies to improve clinical outcome. Whilst surgical resection is a well-established treatment option less than 10% of patients with liver metastases are suitable candidates [4] and there is a need for alternate treatment strategies.

The European Neuroendocrine Tumour Society (ENETS) guidelines highlight various systemic therapies to tackle liver metastases [1]. Whilst recommended as first line systemic therapy, somatostatin analogues improve time to tumour progression but have modest benefits on tumour reduction with the greatest impact in those with a low hepatic tumour burden (< 10%) [5, 6]. Similarly, peptide receptor radionuclide therapy (PRRT) with [^177^Lu]Lu-DOTATATE demonstrates significant improvement in clinical outcomes but has reduced impact in those where liver lesions are greater than 30 mm attributable to the short, soft-tissue penetration depth, 2.2 mm, of [177Lu] [7]. There has been much interest therefore, in liver directed therapies.

Selective internal radiotherapy therapy (SIRT) allows the selective cannulation of the hepatic arteries followed by administration of radioactive microspheres incorporating radionuclides yttrium-90 ([^90^Y]) or holmium ([^166^Ho]) directly to metastases vasculature. SIRT allows the selective delivery of high dose radiation to the cancer with minimal embolic effect. Evidence supporting SIRT in NENs primarily stems from small retrospective studies, including a recent meta-analysis of 27 studies that reported a collective response of 51% and disease control rate of 88% with a median overall survival (OS) of 32 months [8]. Based on the available body of predominantly retrospective evidence, SIRT is recommended for the management of liver metastases from NEN [9]. However, there is a paucity of prospective studies which are needed to effectively guide patient therapy.

For HCC, personalised dosimetry (specific absorbed dose to tumour) has been shown to be associated with superior clinical outcomes compared to standard dosimetry, such that a minimum target tumour dose of 205 Gy is associated with favourable outcomes [10–12]. Personalized dosimetry uses the Tc-99 m macro-aggregated albumin SPECT/CT scan (Tc-99 m MAA) that is routinely performed [13], to predict the distribution of [^90^Y] microspheres between normal liver tissue and tumour. Evaluation of the Tc-99 m-MAA images enables personalised activity prescription that optimises the absorbed tumour dose while ensuring that safety thresholds for healthy lung and liver exposures are not exceeded. This approach allows for a more accurate prediction of clinical outcomes, although the distribution of Tc-99 m-MAA is a pretreatment estimation of [90Y] microspheres [11, 14, 15]. While, the estimation is predictive, verification of post-SIRT distribution of [90Y] is necessary to provide an accurate assessment of absorbed dose either with bremsstrahlung SPECT/CT or PET/CT. The overall aim of this prospective study was to assess the efficacy and safety of SIRT using TheraSphere in patients with liver dominant disease from NEN. In addition, dose-response relationship was explored for both the tumour and perfused normal liver.

## Methods

### Study design and participants

The ArTisaN study was a phase 2, open-label study, with patients recruited from Hammersmith Hospital, Imperial College Healthcare NHS Trust and University Hospitals Birmingham NHS Foundation Trust. Eligible patients were at least age 18 with unresectable liver only or liver predominant metastatic disease, with histologically confirmed NEN. Metastases within the liver occupied *≥* 25% but less than < 60% of the normal hepatic parenchyma. Limited extra-hepatic disease including lung nodules and mesenteric or portal lymph nodes ≤ 2.0 cm were permitted. Patients were required to have received at least one previous line of therapy including SSA. All patients had to have an ECOG performance status of 0–1 and normal liver function. Patients who had previously received TAE, TACE or SIRT were excluded. The full inclusion and exclusion criteria have been previously published [16]. Suitability for SIRT was discussed in a central neuroendocrine multidisciplinary meeting.

### Procedures

Patients underwent screening including blood tests, QoL questionnaires (EORTC QLQ-C30 and EORTC QLQ-GINET21) and tumour imaging; CT scan of the chest, abdomen (triple phase), and pelvis. Pre-treatment angiography was performed to assess tumoural vasculature and any aberrant vessels were coiled at the time of angiography. After Tc-99 m-MAA administration a SPECT/CT scan was performed to determine the hepatopulmonary shunt fraction and extra hepatic deposition. A lung shunt > 20% and no correctable extra hepatic deposition precluded treatment. Perfused volume was determined using the anatomic delineation (CT-scan) and was either the whole liver volume (bifocal) or lobe volume (unifocal). The required activity was determined using a single compartment model, and the Medical Internal Radiation Dose (MIRD) formalism with a target dose of 120 Gy [14]. TheraSphere SIRT was planned within two weeks of baseline angiography.

Prior to SIRT, patients received 24-hour infusion of octreotide (100 mcg/hr) to protect against carcinoid crisis both prior to and following definitive SIRT. In patients with bifocal disease, a staged approach was undertaken where the lobe with the greatest tumour burden targeted first, and the subsequent lobe treated 4–6 weeks later. SIRT was administered by a single interventional radiologist (RT) with over 10 years of experience in SIRT administration at either Hammersmith Hospital or The London Clinic. Following treatment, patients received a reducing dose of dexamethasone, proton pump inhibitor and ursodeoxycholic acid to mitigate against any gastric and hepatic complications as per local protocol. Bremsstrahlung [^90^Y] SPECT/CT imaging was performed either on the day or the day after TheraSphere administration. Bremsstrahlung SPECT/CT imaging was performed on a Intevo Bold (Siemens Healthcare) machine. Images were acquired on a 128 × 128 matrix. SPECT scan parameters were as follows: Number of frames 64, angle of rotation 180° per head (total angle of rotation 360°), energy window 71–119 keV, reconstruction 3D OSEM using 8 iterations, 8 subsets, and Gaussian filter with 9.00 mm FWHM. Retrospective post therapy dosimetry was performed using the Bremsstrahlung [^90^Y] SPECT/CT imaging. Perfused liver and tumour segmentation were conducted by an independent Interventional Radiologist with over 40 years of experience (PT) and dose calculations being performed by a Nuclear Medicine Physicist (PK) using Simplicit90Y dosimetry software (Mirada Medical, USA). Patients were reviewed at week 8 and every 3 months thereafter until disease progression, withdrawal from trial or death occurred. Safety bloods, imaging for response assessment and QoL assessments were undertaken at each time point.

### End points

The primary end point for the study was the ORR in the treated liver volume, determined as either complete or partial response, as per RECIST 1.1 and mRECIST at 6 months [17]. Response was assessed by two independent radiologists with over 20 years’ experience (RT and AA), one blinded to clinical outcome (AA). Primary safety end point was the incidence and causality of AEs using the National Cancer Institute – Common Toxicity Criteria (NCI-CTC version 5) at 6 months.

Secondary end points were progression free survival (PFS) and liver progression free survival and overall survival (OS). PFS was defined as the time from treatment to first documentation of disease progression determined by RECIST 1.1. OS was defined as time from treatment to death from any cause. QoL was assessed using QLQ C-30 and GI.NET21 administered at baseline, 8 weeks, 3 and 6 months. Changes in chromogranin A were assessed at these time points with biochemical response defined as a reduction of 50% or more in chromogranin A.

### Statistical analysis

Patients who had received treatment and had had at least one response assessment following SIRT were included for response assessment (per protocol analysis). All patients who had received at least one SIRT were included in the safety analysis. The frequency and grade of adverse events was described.

A sample size of 21 was determined to give an estimated ORR of 40% with an alpha of 3.7% and power of 95%. An additional 8 patients were recruited to account for screen failures and drop-outs. Only those patients having week 8 assessments were included in the per-protocol analysis. ORR was defined as the number of complete and partial responses according to RECIST 1.1 or mRECIST. The absorbed dose to the whole liver and tumour was derived and correlated with response (RECIST 1.1 or mRECIST) and survival outcomes. Median hepatic specific PFS, PFS and OS were determined using Kaplan-Meier statistics. QoL questionnaires were analysed in accordance with the EORTC scoring manual. All analysis was conducted using SPSS version 27.0 (IBM).

## Results

**Fig. 1:**
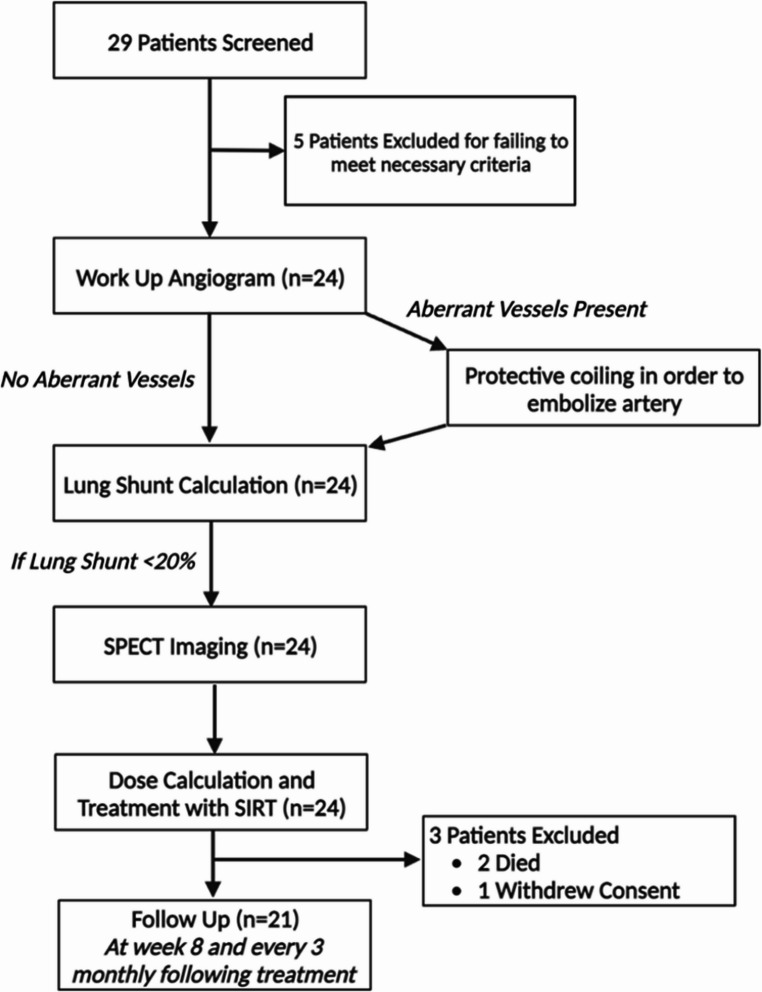
Flowchart summarising the trial protocol. SPECT = single-photon emission computed tomography, SIRT = selective internal radiation therapy

**Fig. 2:**
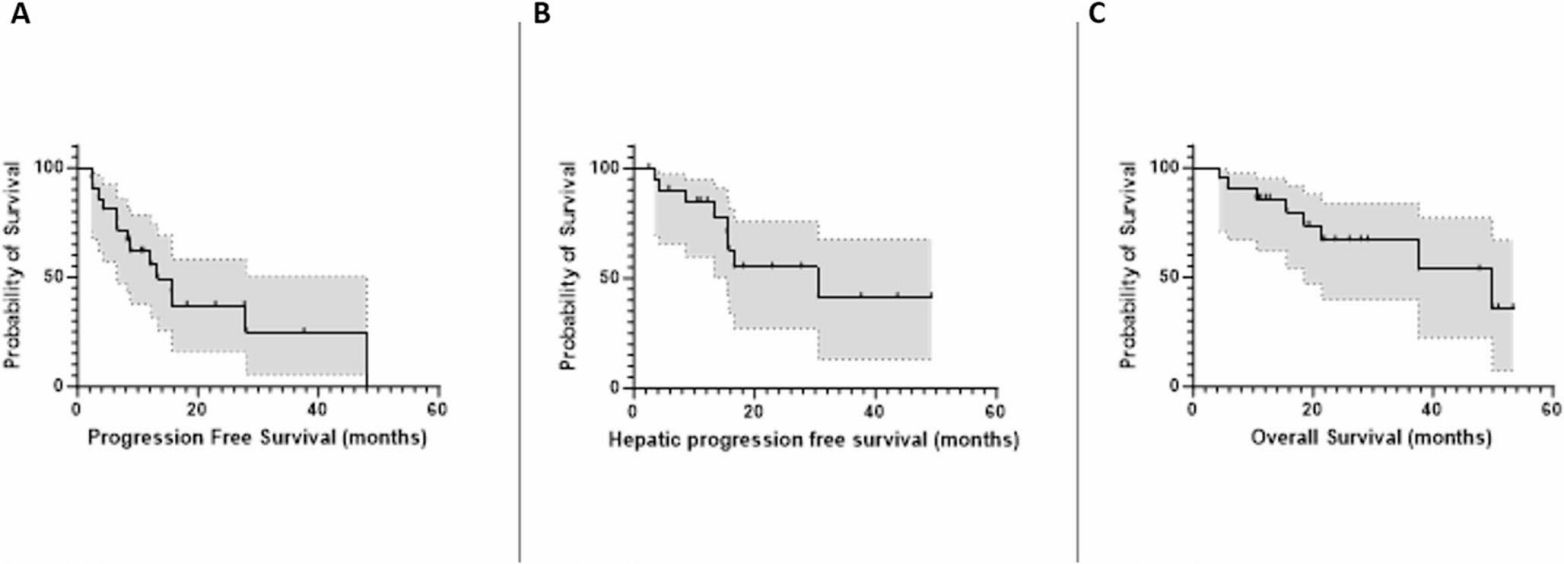
Progression free survival (**A**), hepatic specific progression free survival (**B**) and overall survival (months) (**C**) in patients receiving SIRT with TheraSphere for metastatic NEN

From February 2019 to November 2022, 29 patients consented. Following screening, five patients did not meet inclusion criteria, and one patient withdrew consent. A further two patients died within a month of work-up from progressive disease and did not receive SIRT. Twenty-two patients were treated and 21 completed the week-8 assessment and were included in the per protocol analysis (Fig. 1). The median age of the study cohort was 63years, with the most common site of primary disease being the small bowel (48%). Most patients had grade 2 disease (*n* = 14, 67%) and six (29%) patients had extrahepatic disease. Patients were heavily pretreated with eleven patients (52%) having had at least two previous lines of systemic therapy including seven patients who had received PRRT prior to SIRT. Two patients had functional tumours with symptoms managed with lanreotide. Baseline characteristics are shown in Table 1. The median lung shunt fraction was 3.5% (SD *±* 2.4). Five patients received single lobar SIRT whilst the remaining underwent sequential therapy, the median activity administered to the left lobe was 1.9 GBq (SD *±* 1.0) and 3.7 GBq (SD *±* 1.3) for the right lobe. At 8 weeks, ORR was 14% (*n* = 3) according to RECIST 1.1 and 45% (*n* = 9) according to mRECIST. Of interest, 15 (71%) patients had SD at 8 weeks according to RECIST 1.1, seven of whom had PR when using mRECIST. One patient developed new lesions within the liver outside of the treated liver volume and two patients developed new extra-hepatic metastatic disease. At month-3, ORR were 10% and 44% according to RECIST1.1 and mRECIST respectively (*n* = 17); at month-6, ORR were 8% and 20% according to RECIST1.1 and mRECIST respectively (*n* = 12) (Table 2). We then considered both overall and hepatic specific PFS. The median hepatic specific PFS was 48.1months (95%CI: unable to be calculated) (Fig. 2A) and median PFS was 13.3months (95%CI: 7.0–19.7) (Fig. 2B) suggesting that SIRT resulted in good control of hepatic disease. The OS was 49.9months (95%CI: 18.4–81.5) (Fig. 2C).

**Table 1:** Baseline characteristics of the per-protocol population (*N* = 21)

Mean (*±* SD) Age (Years)	64.1 (11.9)
Sex	
Male	9 (43%)
Female	12 (57%)
Primary Tumour	
Small Bowel	10 (48%)
Pancreas	4 (19%)
Rectal	2 (10%)
Lungs	2 (10%)
Unknown	2 (10%)
Bile Duct	1 (5%)
ECOG Performance Status	
0	10 (48%)
1	11 (52%)
Grade	
1	1 (5%)
2	14 (67%)
3	3 (14%)
Unknown	2 (10%)
Extrahepatic Disease	
Yes	6 (29%)
No	15 (71%)
Previous Treatments	
Somatostatin Analogue	19 (90%)
Surgery	7 (33%)
Peptide Receptor Radionuclide Therapy	6 (29%)
Chemotherapy	6 (29%)
Everolimus	3 (14%)
Median (IQR) Serum Chromogranin A Level (µg/L) at Baseline	114.5 (722)
Median (IQR) Bilirubin Level at Baseline (µmol/L)	11 (7)
Selective Internal Radiation Therapy Details	
Mean (SD) Liver Volume (cc)	638 (398)
Left Lobe	1330 (488)
Right Lobe	3.45 (2.42)
Median (SD) Lung Shunt Fraction (%)	
Mean (SD) Administered Activity (GBq)	
Left Lobe	1.86 (1.01)
Right Lobe	3.66 (1.30)

**Table 2:** Response assessment according to RECIST 1.1 and mRECIST at 8 weeks, 3 and 6 months

	Week 8		3 Months		6 Months	
	Liver-specific response	Patient-based response	Liver-specific response	Patient-based response	Liver-specific response	Patient-based response
RECIST 1.1	*N***= 21**		*N* **= 17**		*N***= 12**	
Complete Response	0	0	0	0	0	0
Partial Response	3 (14%)	2 (10%)	2 (10%)	1 (5%)	1 (8%)	1 (8%)
Stable Disease	18 (86%)	16 (76%)	13 (76%)	13 (76%)	11 (92%)	11 (92%)
Progressive Disease	0	3 (14%)	1 (5%)	3 (14%)	0	0
mRECIST	*N***= 20**		*N***= 16**		*N***= 10**	
Complete Response	0		0		0	
Partial Response	9 (45%)		7 (44%)		2 (20%)	
Stable Disease	11 (55%)		9 (56%)		8 (80%)	
Progressive Disease	0				0

Median chromogranin A levels decreased significantly from 114.4 pmol/L (IQR 33–754) at screening to 86 pmol/L (IQR 30–195) at week 8 (*p* = 0.01), and remained stable at 3months, 83 pmol/L (IQR 30–195) and 6months, 75 pmol/L (IQR 24–263) (supplementary Fig. 1). No correlation was observed between ORR and changes in chromogranin levels.

The population assessed for safety consisted of 21 patients. The most frequent clinical toxicity was grade 1 fatigue experienced by 13 patients (62%) and grade 1 abdominal pain in 9 (43%) patients (Table 3). Two patients (10%) developed grade 3 radioembolisation induced liver disease (REILD) which resolved without sequelae with steroids. Both patients had normal liver function tests at baseline. One patient had had previous therapy with SSAs, CAPTEM and [^177^Lu]Lu-DOTA-TATE albeit 9 months prior to receiving SIRT. The other patient was in receipt of concurrent SSAs. Both received bilobar treatment. In terms of laboratory toxicities, the most commonly reported event was grade 3 lymphocytopenia, experienced by four (19%) patients that resolved without sequelae. No laboratory toxicity above grade 3 was seen (Table 4). No patient experienced symptoms of hormone excess following SIRT.

**Table 3:** Adverse events according to NCI CTC version 5.0

	Grade 1	Grade 2	Grade 3
Anorexia	6 (29%)	3 (14%)	0
Nausea	1 (5%)	5 (23%)	0
Vomiting	0	1 (5%)	0
Flushing	5 (23%)	1 (5%)	0
Fatigue	13 (62%)	7 (33%)	0
Abdominal Pain	9 (43%)	3 (14%)	0
Bloating	5 (23%)	1 (5%)	0
Other			
Arterial Plug Migration			1 (5%)
Radiation Induced hepatitis			2 (10%)

All treatment-related clinical toxicities reported within the first 6 months following treatment

Severity determined via National Cancer Institute – Common Toxicity Criteria for Adverse Events version 5.0

**Table 4:** Laboratory toxicities across study duration according to NCI CTC version 5.0

	Baseline		Week 8		3 Months		6 Months	
	Grade 1	Grade 2	Grade 1-2	Grade 3	Grade 1-2	Grade 3	Grade 1-2	Grade 3
Creatinine Increase	1 (5%)	0	0	0	1 (5%)	0	0	0
Bilirubin Increase	0	0	0	1 (5%)	0	1 (5%)	0	0
ALP Increase	2 (10%)	0	6 (29%)	0	7 (33%)	0	4 (19%)	0
AST Increase	6 (29%)	0	5 (24%)	0	3 (14%)	0	2 (10%)	0
ALT Increase	0	0	1 (5%)	0	0	0	0	0
Low Albumin	0	0	2 (10%)	0	0	0	0	0
Anaemia	3 (14%)	0	2 (10%	0	0	0	0	0
Neutropenia	1 (5%)	2 (10%)	0	0	0	0	0	0
Lymphocytopenia	7 (33%)	0	7 (33%)	3 (14%)	0	1 (5%)	2 (10%)	0
Thrombocytopenia	3 (14%)	0	1 (5%)	0	3 (14%)	0	0	0

Severity determined via National Cancer Institute – Common Toxicity Criteria for Adverse Events version 5.0

*ALP* Alkaline Phosphatase, *AST* Aspartate Aminotransferase, *ALT* Alanine Aminotransferase

**Fig. 3:**
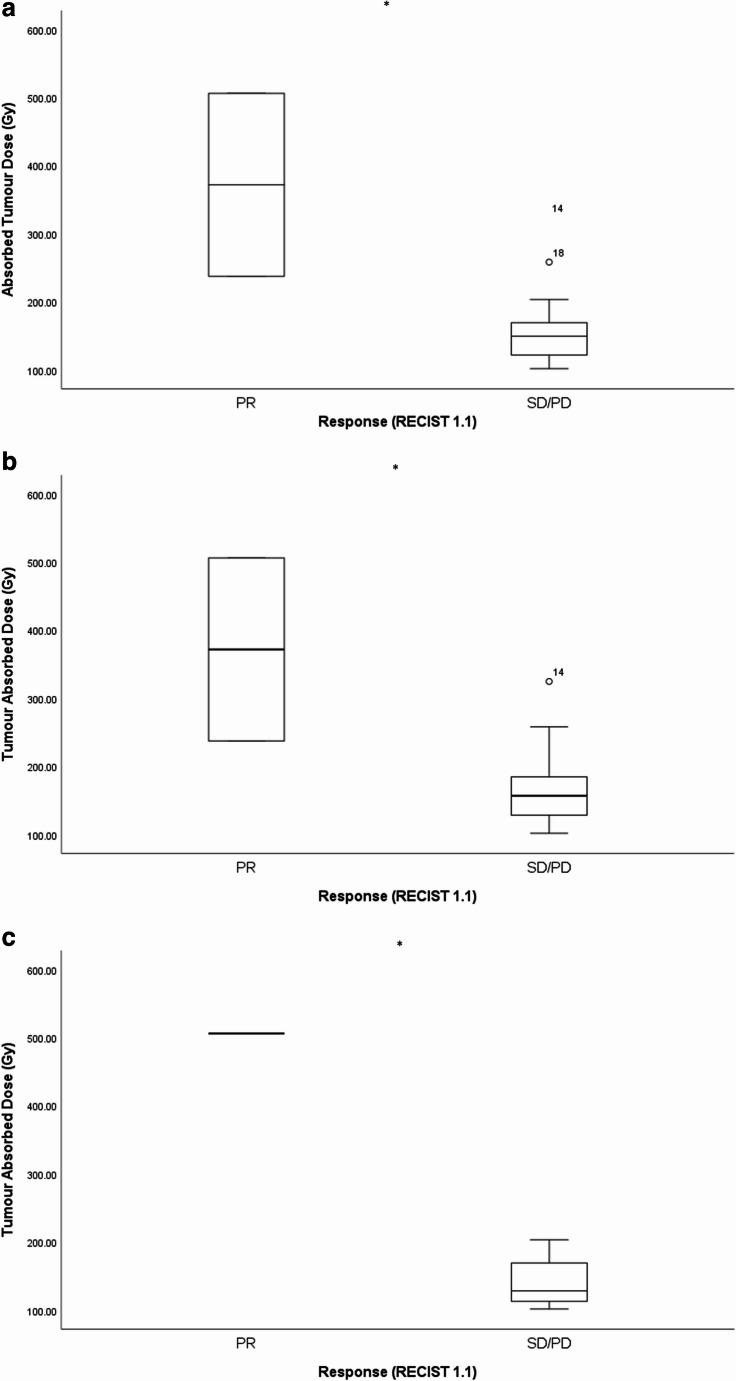
Bar graphs illustrating a significant relationship between tumour absorbed dose and response (RECIST 1.1) using Anova at 8 weeks (**A**), 3months (**B**) and 6 months (**C**). * *p* < 0.05, PR partial response, SD stable disease, PD progressive disease

Post treatment dosimetry was evaluated in all treated patients. The median dose delivered to right sided tumours was 148 Gy (IQR 113–184 Gy) and a median of 125 Gy (IQR 94–128 Gy) was delivered to left sided tumours; the median total tumour absorbed dose was 153 Gy (IQR 128–354 Gy). When considering the relationship between total tumour absorbed dose and response (RECIST 1,1 and mRECIST), ORR was associated with increased median absorbed dose at each time point (Fig. 3A-C).

**Fig. 4:**
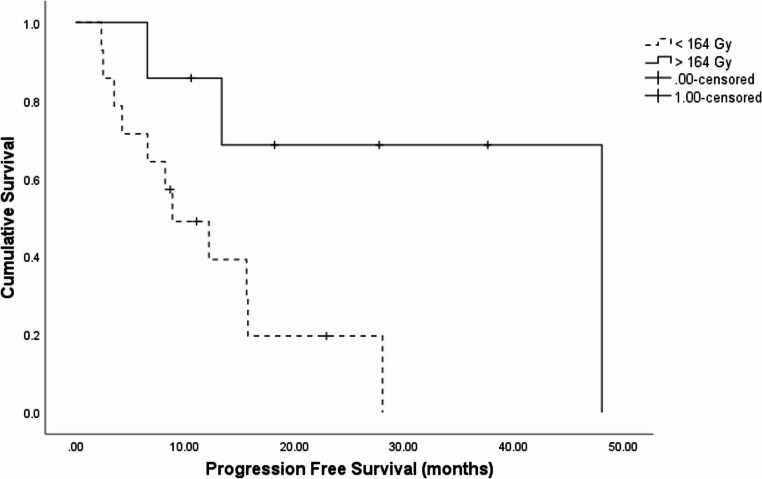
Kaplan Meier analysis illustrating improved progression free survival (months) on receipt of > 164 Gy tumour absorbed dose

In patients with tumour response at 8 weeks the mean tumour absorbed dose was 372 *±* 190 Gy compared with 159 *±* 55 Gy in non-responders (*p* < 0.001). In patients with tumour response at 3months, mean absorbed dose in responders was 372 *±* 190 Gy compared with 173 *±* 60 Gy in non-responders (*p* = 0.004). In patients with tumour response at 6months, mean absorbed dose was 506 *±* 53 Gy in responders compared with 142 Gy in non-responders (*p* < 0.001). In the ROC analysis, Youden’s J gave a tumour absorbed dose of 164 Gy to have the highest sensitivity and specificity in determining ORR at 3months, 67% and 100%, respectively. The area under the curve was 0.8 (95%CI: 0.6–0.9). When using this cut-off, a total tumour dose of > 164 Gy was associated with improved PFS (median PFS 48.1months compared to 8.8months, HR 0.2, 95%CI 0.05–0.96. *p* = 0.03) (Fig. 4). We did not observe a relationship between perfused normal liver absorbed dose and toxicity. In the two patients that developed REILD, an absorbed dose of 80 Gy and 101 Gy were delivered to the perfused normal liver. All patients received less than 150 Gy to the whole liver consistent with the product use (median 113.8 Gy, IQR 92–117 Gy) and the median absorbed dose to the total perfused volume was 101.4 Gy (IQR 94–133 Gy) [13].

**Fig. 5:**
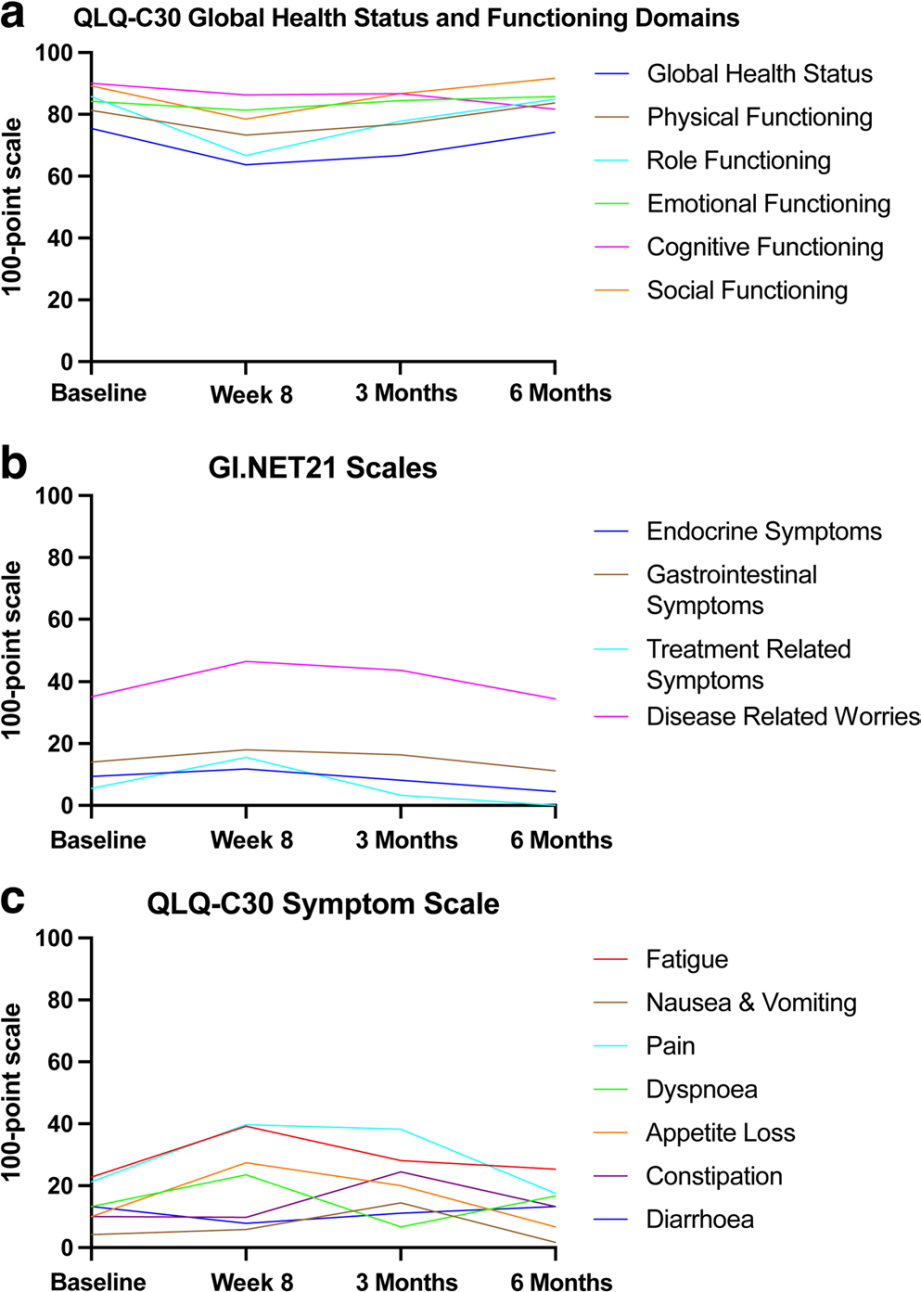
Quality of life assessment via european organisation for research and treatment of cancer questionnaires. (**A**) QLQ C-30 global health status and functioning domains at baseline, week 8, 3 months and 6 months., (**B**) GI.NET21 scales at baseline, week 8, 3 months and 6 months., (**C**) QLQ-C30 symptom scales at baseline, week 8, 3 months and 6 months

Analysis of functioning scales, symptom scales and global health status using the QLQ-C30 and GI.NET21 questionnaires were completed by all but one patient during the follow-up period. When considering global health status, this reduced at 8 weeks (*p* = 0.06) and 3months returning to baseline at 6 months (Fig. 4A). No significant decline or improvement in physical, emotional, cognitive, role and social functioning was observed during the study period (Fig. 5A). No deterioration in NET symptoms was noted following SIRT administration (Fig. 5B). In accordance with the reported AE data, the main symptom experienced by patients using the QLQ-C30 symptom scale, was fatigue but no significant change was noted from baseline (Fig. 5C).

A post-hoc analysis was conducted of those patients who had previously received [^177^Lu]Lu-DOTA-TATE (*n* = 7). The mean length of time between previous [^177^Lu]Lu-DOTA-TATE and SIRT was 3.2years (range 0.7–6.3years). One patient experienced grade 3 REILD and two experienced transient grade 3 lymphopaenia at 8 weeks. No other additive toxicities were observed. In terms of response, one patient experienced PR with additional SIRT whilst the others had SD at 6months. An additional two patients received [^177^Lu]Lu-DOTA-TATE following SIRT both of whom experienced SD within the liver 6months following the completion of PRRT.

## Discussion

To our knowledge, this is the first prospective study assessing the efficacy of SIRT using TheraSphere in patients with unresectable liver metastases from NEN. We have demonstrated a favourable ORR and a median hepatic PFS of greater than 2 years in a heavily pre-treated cohort with an acceptable toxicity profile. Importantly, we did not observe any significant deterioration in QoL following treatment. Consistent with the published literature on personalised dosimetry, we observed a positive relationship between tumour absorbed dose and response to SIRT. Whilst SIRT is included in international guidelines for the management of liver metastases from NEN [9] these recommendations are based predominantly on meta-analyses of retrospective or institutional case series. We have provided much needed additional prospective evidence supporting the use of SIRT for the management of liver metastases secondary to NEN.

Existing therapeutic strategies for the management of liver metastases, including TAE/TACE and systemic agents, are associated with significant side-effects and variable response rates [9]. In particular, the use of TAE/TACE is associated with a significant risk of post-embolization syndrome and carcinoid crisis [18]. This is reflective of the larger experience with TAE/TACE versus SIRT in other tumour types [19, 20].

Our results highlight the low incidence and severity of treatment-related toxicities consistent with previous studies of SIRT [21, 22]. We report two cases of REILD, a well-documented side-effect of [90Y] therapy both of which resolved with medical treatment. We did not, as others have observed, note any cases of portal hypertension, liver fibrosis and cirrhosis [23, 24]. These are important long-term toxicities that are gaining increasing importance in the management of NEN given the long prognosis although a recent study suggests that the incidence of these complications is comparable to that for TACE [25].

The most pertinent finding from our work is the long hepatic specific PFS of 48 months. This is despite our cohort receiving a median of two prior lines of therapy. We report OS and PFS consistent with large retrospective cohorts, despite our trial comprising of a significantly higher proportion of patients with grade 2–3 disease [22, 26, 27]. Braat and colleagues report an ORR of 20% and an OS of 2.6 years in 244 patients treated with SIRT [26]. Similarly, the ReSIN registry in 170 patients report an ORR of 36% and median OS of 33 months [22]. Of interest, in both of these retrospective studies the majority of patients were treated with resin sphere and dosed using the BSA method. We performed a retrospective analysis of tumour absorbed dose using Bremstrasstrahlung SPECT/CT post therapy imaging. Whilst not as accurate as post-treatment positron emission tomography in assessing dosimetry [28], SPECT has been used extensively to assess tumour absorbed dose and has been shown to predict clinical outcome to SIRT. In our study, a median cumulative tumour dose of 153 Gy was delivered which is similar to the dose reported in previous retrospective studies in NEN [29]. Moreover, whole liver absorbed dose was below current recommendations of 120 Gy resulting in minimal toxicities [11–13]. It is well established that the use of personalised dosimetry results in improved clinical outcomes with an optimal tumour dose of at least 200 Gy being associated with improved clinical outcomes in hepatocellular carcinoma [12, 13]. The optimal minimal absorbed dose to be delivered in NENs is yet to be determined. There are only three, small retrospective studies in NENs investigating the tumour dose-response relationship, two studies that use [^90^Y] and another that investigated [166Ho]. These studies confirm our findings that higher tumour absorbed dose corresponds to improved ORR with a minimum tumour absorbed doses associated with response [15, 29, 30]. Consistent with these studies, we illustrated that a tumour absorbed dose of above 164 Gy was associated with improved PFS supporting the role for a personalised dosimetry approach in NENs.

We performed a post-hoc analysis in those patients who had previously received PRRT. The recent HEPAR plus study illustrated the efficacy and safety of the addition of SIRT using holmium microspheres following PRRT [31]. Given the relative depth of penetration of yttrium and lutetium, the combination therapy holds significant promise in the management of bulky disease. Whilst the median interval between PRRT and SIRT in our cohort was long, we noted no additional toxicity in this subgroup with response being similar to the remainder of the cohort supporting the use of this combination moving forward.

A key strength of our study is the prospective evaluation of QoL in patients undergoing radioembolisation. QoL is gaining increasing importance in oncology studies particularly as the key goal of treatment in these patients is palliation. Only one other study evaluated QoL in patients prospectively undergoing SIRT [30]. Consistent with our findings, the authors did not observe any significant deterioration in QoL outcomes 3 months following SIRT. Similarly, we did not observe any improvement in QoL which may be attributable to the lack of baseline symptoms prior to patients receiving SIRT with only two patients having carcinoid symptoms. Our QoL findings are concordant with our AE data, with fatigue and pain being the highest scored items on the QLQ-C30 symptom scale. Taken with the response and survival data, this work suggests that SIRT is a viable option for those with NEN liver metastases.

There are multiple strengths to our study including its prospective design and use of robust and evidence-based criterion to measure outcomes. However, it is not without limitations the most important being the prescription methodology for the delivery of SIRT. This study used a prescription of 120 Gy to the perfused volume which was used routinely at the time of the study initiation. Most centres now use personalised dosimetry as standard which further optimises the treatment by using tumour and normal liver dose thresholds. The use of tumour dose thresholds in an optimised personalised dosimetry approach is attributed to improving outcomes in SIRT [12]. It is possible therefore, that the use of personalised dosimetry may enhance response further and reduce toxicity, which should be explored in future prospective work. Moreover, whilst we used Bremsstrahlung radiation post SIRT to determine tumour-absorbed dose, PET scanning performed ideally the day of SIRT has greater accuracy and should be pursued in future work [32]. Key baseline characteristics varied in our patient cohort, with patients of differing primary tumour sites, ECOG performance status, and tumour grade being enrolled. As these parameters are independent prognostic factors, larger studies that will allow prospective subgroup analyses should be undertaken. Our study conduct was impacted by the Covid-19 pandemic which did result in fewer patients receiving trial related imaging at 6months which may impact on our results.

In conclusion, we have provided much needed, additional prospective evidence demonstrating the efficacy of TheraSphere SIRT supported by dosimetry data. This was accompanied by a favourable toxicity profile with no detriment in QoL. This work further strengthens the position of SIRT in the therapeutic armamentarium for the management of inoperable liver neuroendocrine disease.

## Supplementary information

Below is the link to the electronic supplementary material.

Supplementary File 1 (DOCX 45.4 KB)

## Data Availability

The authors confirm that the data supporting the findings of this study are available within the article.
