# Supplementary material for: Efficacy and evaluation of dose-response relationship of selective internal radiation therapy for the management of liver metastases in neuroendocrine neoplasia
Source: Eur J Nucl Med Mol Imaging. 2026 Feb 17;53(7):4521–31. doi: 10.1007/s00259-026-07762-0 (PMC13197315; doi:10.1007/s00259-026-07762-0)
Supplement: Supplementary file 1 — (DOCX 45.4 KB) [file 259_2026_7762_MOESM1_ESM.docx]

**Supplementary *Figure 1: Levels of tumour marker chromogranin A decreased at each follow-up post-treatment.***

Median values shown, with error bars denoting 75^th^ and 25^th^ percentiles.


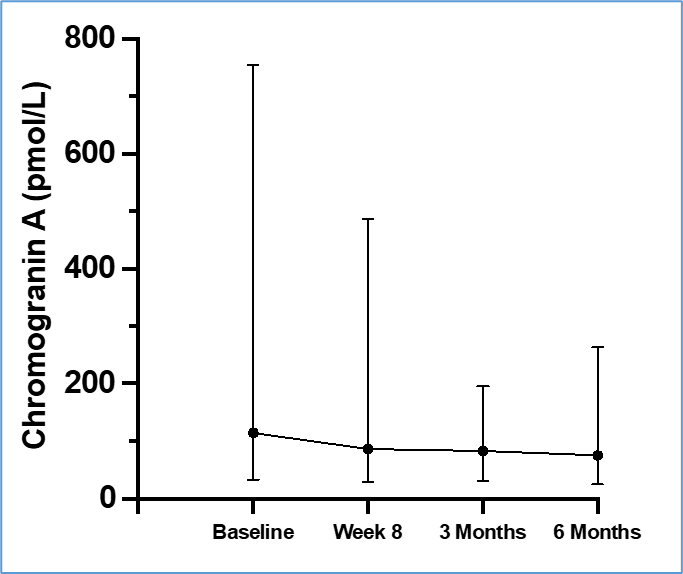


N=18

N=18

N=9

N=6
